# New classification for bone type at dental implant sites: a dental computed tomography study

**DOI:** 10.1186/s12903-023-03039-2

**Published:** 2023-05-25

**Authors:** Shiuan-Hui Wang, Jui-Ting Hsu, Lih-Jyh Fuh, Shin-Lei Peng, Heng-Li Huang, Ming-Tzu Tsai

**Affiliations:** 1 School of Dentistry, China Medical University, Taichung, 404 Taiwan; 2 Department of Biomedical Engineering, China Medical University, Taichung, 404 Taiwan; 3 Department of Dentistry, China Medical University and Hospital, Taichung, 404 Taiwan; 4 Department of Biomedical Imaging and Radiological Science, China Medical University, Taichung, 404 Taiwan; 5 Department of Biomedical Engineering, Hungkuang University, Taichung, 433 Taiwan

**Keywords:** Bone classification, Dental CBCT, Cortical bone thickness, Cancellous bone density, Jawbone

## Abstract

**Objective:**

This study proposed a new classification method of bone quantity and quality at the dental implant site using cone-beam computed tomography (CBCT) image analysis, classifying cortical and cancellous bones separately and using CBCT for quantitative analysis.

**Methods:**

Preoperative CBCT images were obtained from 128 implant patients (315 sites). First, measure the crestal cortical bone thickness (in mm) and the cancellous bone density [in grayscale values (GV) and bone mineral density (g/cm^3^)] at the implant sites. The new classification for bone quality at the implant site proposed in this study is a “nine-square division” bone classification system, where the cortical bone thickness is classified into A: > 1.1 mm, B:0.7–1.1 mm, and C: < 0.7 mm, and the cancellous bone density is classified into 1: > 600 GV (= 420 g/cm^3^), 2:300–600 GV (= 160 g/cm^3^–420 g/cm^3^), and 3: < 300 GV (= 160 g/cm^3^).

**Results:**

The results of the nine bone type proportions based on the new jawbone classification were as follows: A1 (8.57%,27/315), A2 (13.02%), A3 (4.13%), B1 (17.78%), B2 (20.63%), B3 (8.57%) C1 (4.44%), C2 (14.29%), and C3 (8.57%).

**Conclusions:**

The proposed classification can complement the parts overlooked in previous bone classification methods (bone types A3 and C1).

**Trial registration:**

The retrospective registration of this study was approved by the Institutional Review Board of China Medical University Hospital, No. CMUH 108-REC2-181.

## Introduction

The number of patients with missing teeth has been increasing along with the aging population in recent years. The use of dental implants is one of the most common treatment methods for restoring the normal occlusal function of patients with missing teeth [1]. Therefore, how to increase the dental implant success rate is a critical issue. The success rate of dental implants depends on several factors, including the surgeon’s surgical skills, the patient’s postoperative oral hygiene habits, and the thread design or surface treatment of the dental implant. Notably, the bone quality of the jawbone is one of the essential influencing factors [2–6]. Previous literature has reported that good jawbone quality can provide better initial stability, allowing for better osseointegration in future recovery and ensuring a more stable implant, thereby increasing the success rate of dental implant surgeries [7].

The jawbone structure is composed of two layers. Dense cortical bone forms the outer layer, and a porous cancellous bone with trabecular bone structures forms the inner layer. The success rate of dental implant surgery is highly dependent on the jawbone quality, and for its assessment, numerous scholars have proposed various classification methods. These methods can be grouped into three types; in the following sections, they will be referred to as Type I, Type II, and Type III classification methods. Type I refers to Lekholm and Zarb’s jawbone quality classification method and its extensions. At the time of writing, the bone quality classification method they proposed in 1985 [8] is the most widely used. When using this method, the observer subjectively ranks jawbones into four types according to the proportions of cortical and cancellous bone, namely Bone Type 1–Type 4. Several scholars then began using computed tomography (CT) or cone-beam CT (CBCT) to explore this method in greater depth [9–11]. However, the subjective nature of this method and the absence of quantitative analysis may lead to different results depending on the observer. The Type II method is based on the tactile of bone drilling during implant surgery and image Hounsfield unit (HU) for classification and was initially proposed by Misch et al. in 1989 [12]. The Type III method refers to the system that classifies the cortical bone and the cancellous bone separately proposed by Tomaso Vercellotti in 2009 [13]. The lack of quantitative analysis is the con of the two methods mentioned above.

Typically, cortical bone thickness and cancellous bone density are used as quantitative measures for jawbone quality. Many papers have been published on the use of CT and CBCT in measuring cancellous bone density in jawbones [10, 14, 15]. It can be seen from the aforementioned literature that the order of cortical bone thickness and cancellous bone density differs in the four jawbone regions.

Most of the past classification methods of jawbone quality and bone quantity [8–10, 12] have assumed that better bone quality is characterized by thicker cortical bone and denser cancellous bone and that worse bone quality is characterized by thinner cortical bone and less dense cancellous bone. However, our previous study revealed that cortical bone thickness and cancellous bone density are ordered differently in the four jawbone regions, with little correlation between them [16]. As such, this study aimed to propose a new classification method of bone quantity and bone quality at the implant site using CBCT image analysis, classifying cortical and cancellous bones separately, and using CBCT for quantitative analysis. All of the jawbones that may be encountered clinically could be covered by this classification method, thereby providing a reference basis for dentists to classify the jawbone quality before dental implant surgery.

## Materials and methods

### Dental CBCT examinations of patients and implant sites

This study was approved by the Institutional Review Board of China Medical University Hospital, No. CMUH 108-REC2-181. We confirmed that all the methods were performed in accordance with relevant guidelines and regulations. Informed consent was waived by CMUH 108-REC2-181 owing to the retrospective nature of the study. Samples for this experiment were collected for implant-planning purposes.

This retrospective study was conducted at the Dental Division of China Medical University Hospital from August 2018 to March 2020. Promax 3D Max (Planmeca, Helsinki, Finland) was used for dental CBCT imaging, and the scanning parameters were set as follows: voxel size 200 μm, voltage 96 kV, and current 12.5 mA. In this study, 315 suitable dental implant sites recorded from a total of 128 implant patients (66 males and 62 females) were collected, including 42 anterior mandible sites, 127 anterior maxilla sites, 39 posterior mandible sites, and 107 posterior maxilla sites. The study inclusion criteria were as follows: (a) CBCT images were taken before the dental implant surgery, and (b) a dental surgical stent with a radiographic guide was used during the CBCT scan. The exclusion criteria were as follows: (a) CBCT images with motion artifacts due to patient movement during the scanning process, (b) CBCT images with metal artifacts due to the presence of dental implants, amalgam filling, or orthodontic appliances (e.g., bracket, archwire, and miniscrew).

In addition, to prevent factors such as different dental CBCT brands and models from affecting the reproducibility of this experiment, the QRM-MicroCT-HA phantom (QRM GmbH; Moehrendorg, Germany) was also scanned to converse GV to BMD to standardize the research results.

### Measurement of cancellous bone density and cortical bone thickness at the dental implant sites

**Fig. 1:**
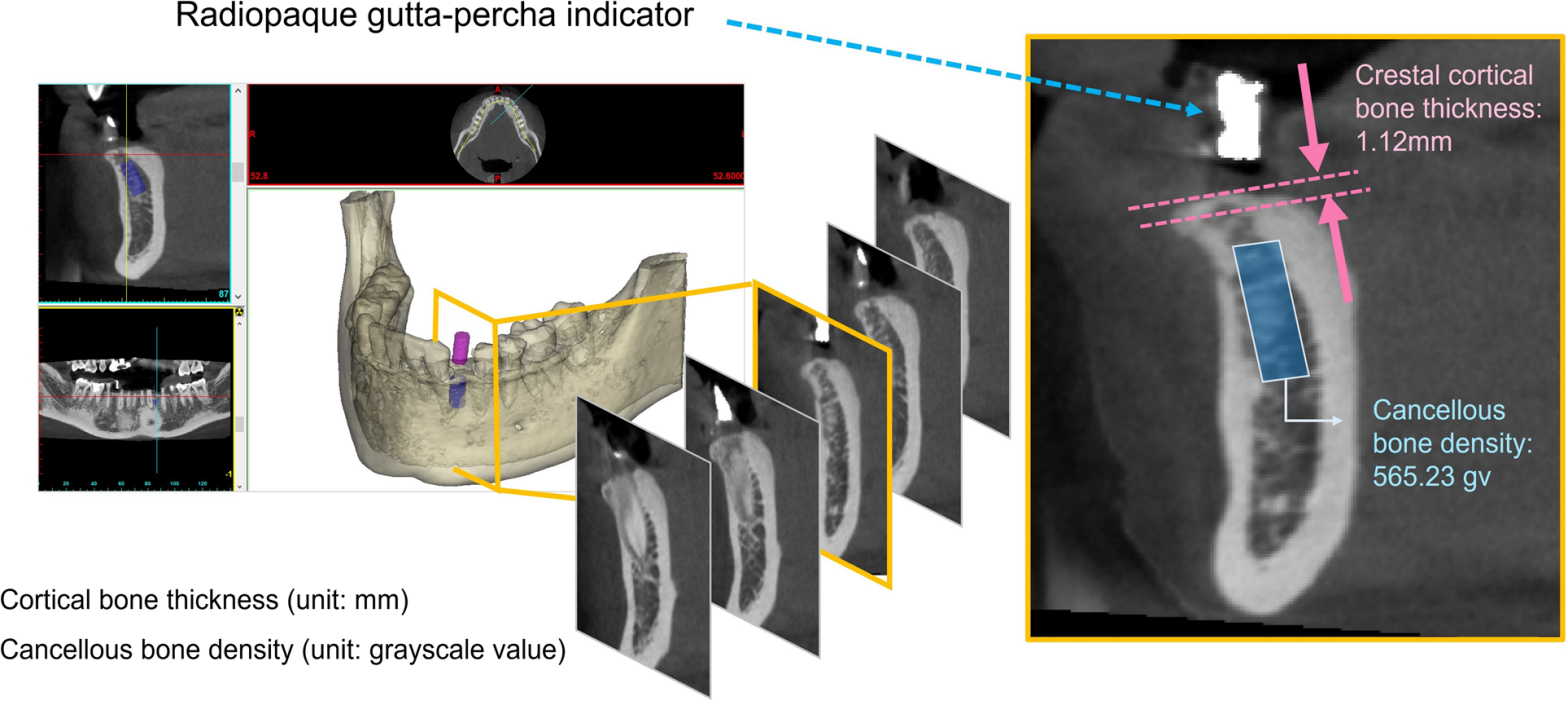
The measurement of cancellous bone density and cortical bone thickness at the dental implant site

CBCT images were input into the medical imaging software Mimics 15.0 (Materialise, Leuven, Belgium) and resectioned along the dental arch to generate orthogonal section images of the potential dental implant sites. All patients underwent CBCT with a dental surgical stent before implantation. According to the radiographic guides (on the dental surgical stent), the referenced information, such as the planned insertion site and angle, was determined. The measurement was performed on a single slice in the center of the radiographic guides, and the thickness measurement was based on the instructions of the radiographic guide on CBCT. The corresponding crestal cortical bone thickness was measured (Fig. 1) in mm. To measure the cancellous bone density, a three-dimensional cylinder simulated the dental implant at the potential implant site, point by the radiographic guides, was created according to the actual implant size (diameter: 3.5, 4.1, 5 mm; length: 10, 11.5 mm). The density of the cancellous bone inside the 3D cylinder was measured on CBCT multi-slices (Fig. 1). The density of bone is expressed by its grayscale value (GV). In addition, the dental CBCT was also used in this study to record images of phantoms with varying bone mineral densities (BMD), thereby establishing the GV/BMD conversion formula. The measurement method for cortical bone thickness and cancellous bone density has been reported in previous literature [10, 14, 16].

### The new classification for bone quality type at the dental implant site of the jawbone

**Fig. 2:**
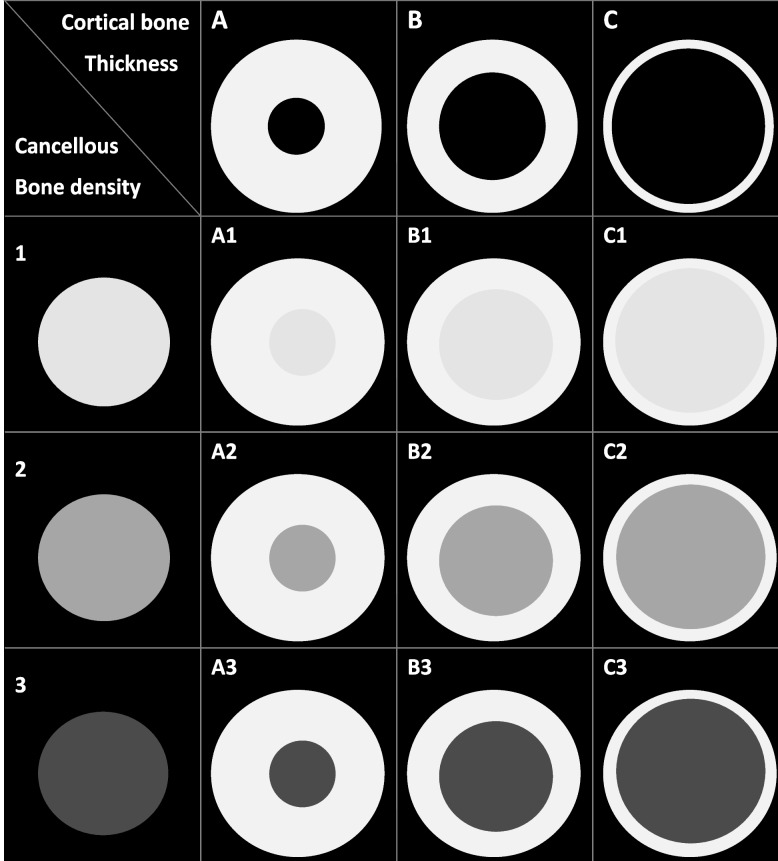
Schematic diagram of the new bone classification; three different thicknesses (**A**, **B**, and **C**) of cortical bone and three different densities (1, 2, and 3) of cancellous bone

First, the cortical and cancellous bones are classified separately; then, according to the measured crestal cortical bone thickness, they are classified into A, B, and C from thick to thin, respectively. The cancellous bone density is classified into 1 (high density), 2 (intermediate density), and 3 (low density). Category 1 is lighter, representing more radioopacity, and category 3 is darker, indicating more radiolucency. The corresponding numerical range for each level will be determined by the final measurement results. Finally, through the permutation and combination of the crestal cortical bone thickness and cancellous bone density, the jawbones are divided into a total of nine bone types (Fig. 2): A1, A2, A3, B1, B2, B3, C1, C2, and C3. Among them, A1 represents the jawbone with the thickest cortical bone and the densest cancellous bone, while C3 represents the one with the thinnest cortical bone and the least dense cancellous bone.

### Statistical analysis

Measurement accuracy before measuring cortical bone thickness and cancellous bone density was validated. Intraclass correlation coefficients (ICCs) were calculated to determine the reliability of the intra- and inter-examiner measurements. Twelve CBCT image data were randomly selected from 315 implant sites to assess intra- and inter-examiner error. Two experienced examiners, a dentist with 15 years of experience and a dental radiation technician with five years of experience interpreting dental CBCT images, were recruited to test the intra-observer agreement measuring the cortical bone thickness and cancellous bone density.

The results of this study involved descriptive statistical analysis by determining the mean value, standard deviation, and proportion of each bone type in all of the samples. The data were also grouped into the following regions: anterior maxilla, posterior maxilla, anterior mandible, and posterior mandible. The results were discussed from two aspects: (a) the percentages of each of the nine bone types for the whole jawbone based on the new bone classification system; and (b) the percentages of bone types based on the four jawbone regions. In addition, a normal distribution analysis of the Kolmogorov–Smirnov test for cortical bone thickness and cancellous bone density was performed. Statistical Package for the Social Sciences (IBM Corporation, Armonk, NY, USA) was used for all statistical analyses.

## Results

### Distribution of cortical bone thickness and cancellous bone density at the dental implant sites and the new bone classification

To calculate the inter-examiner error, cortical bone thickness, and cancellous bone mineral density in CBCT, two examiners measured each of the factors once, and the ICC values were found to be 0.986 (cancellous bone) and 0.959 (cortical bone). To account for intra-examiner error, cortical bone thickness concavity and cancellous bone mineral density in CBCT were measured twice by a single examiner, and the ICC values were found to be 0.989 (cancellous bone) and 0.968 (cortical bone). These values suggest that the method's intra- and inter-inspector errors were negligible in this study.

**Fig. 3:**
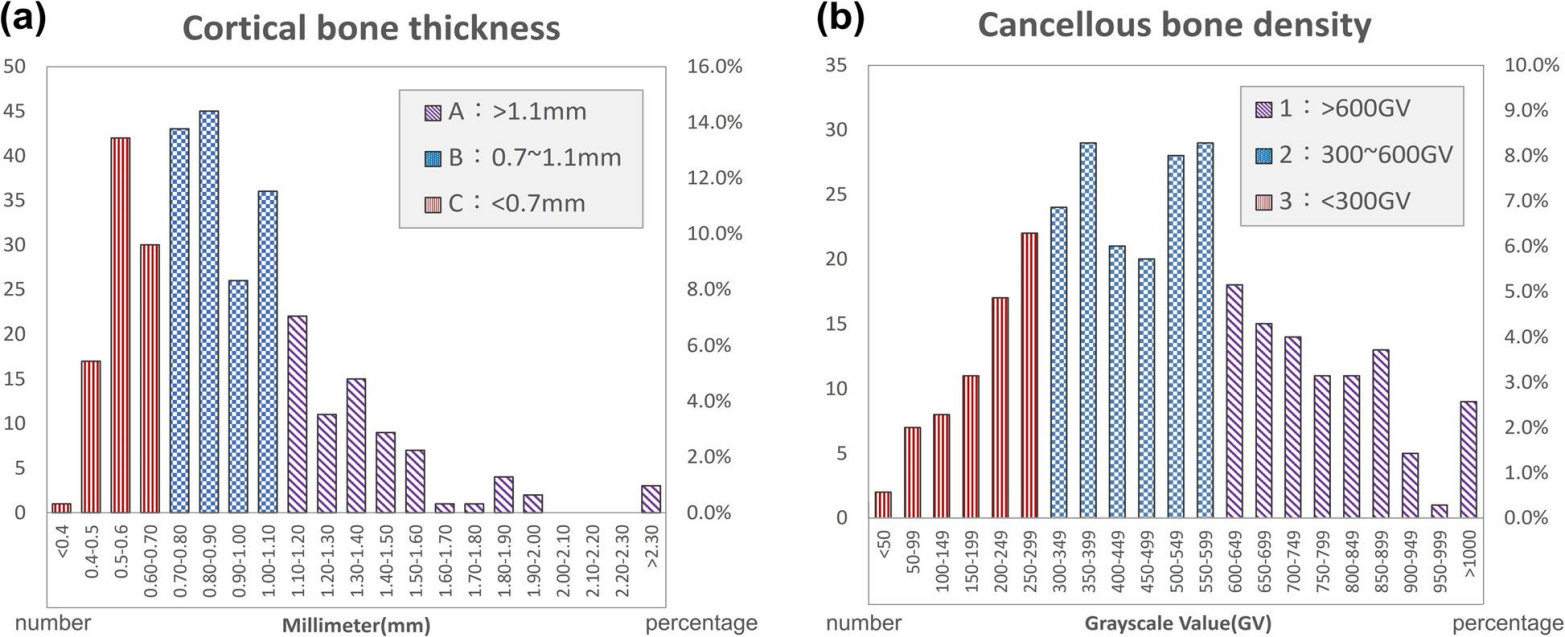
**a** The distribution of cortical bone thickness. (A: red, B: blue, C: purple); **b** The distribution of cancellous bone density (1: red, 2: blue, 3: purple)

**Fig. 4:**
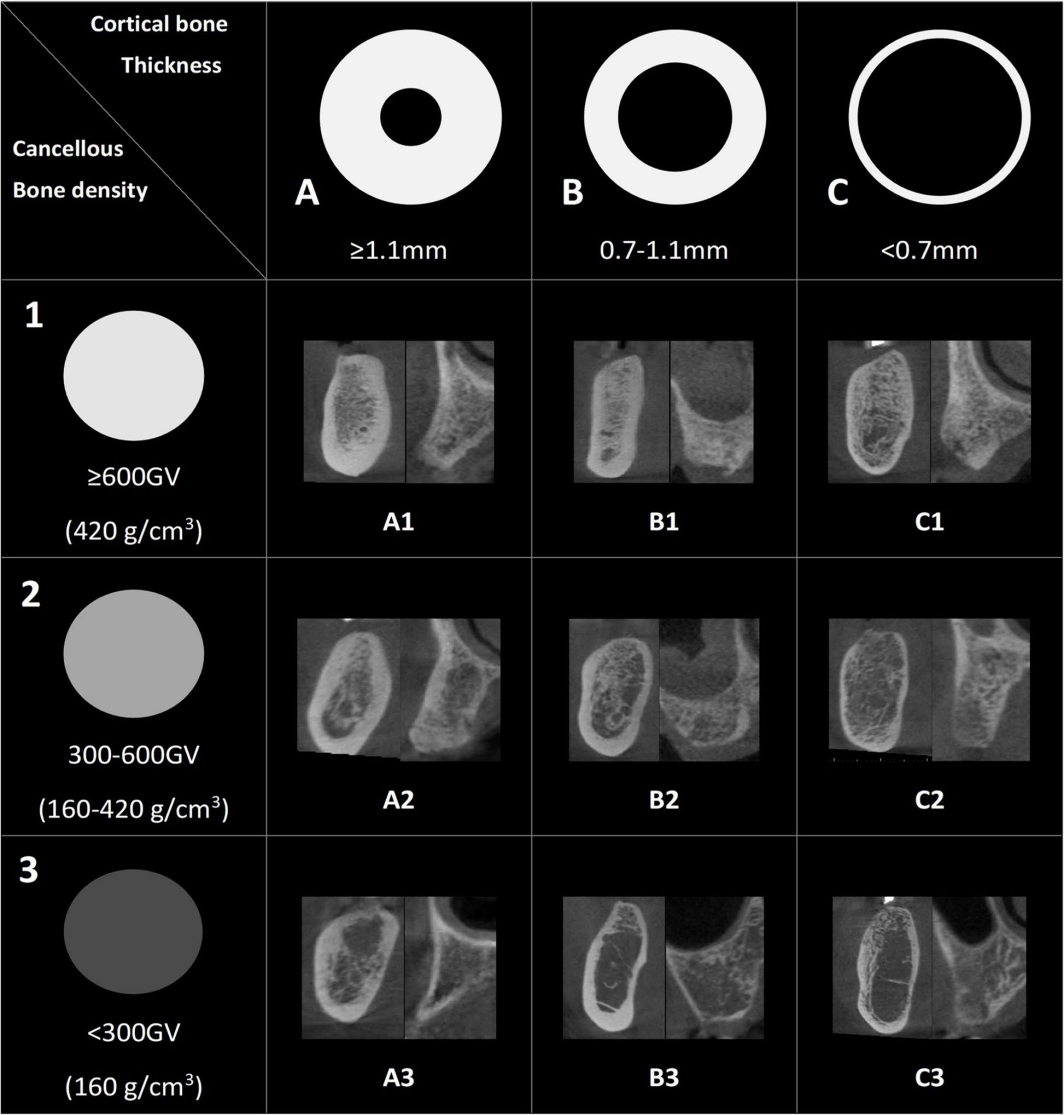
Example of the nine bone types based on the new bone classification

According to the results of this experiment, in the normal distribution analysis of the Kolmogorov–Smirnov test, cancellous bone density showed a normal distribution (*p* > 0.05), and cortical bone density showed an abnormal distribution (*p* < 0.05), as shown in Fig. 3. Based on the new jawbone classification system proposed in this study (Fig. 4), the crestal cortical bone thickness of the jawbone was classified into three levels: A, B, and C, with a classification interval of A: > 1.1 mm, B: 0.7–1.1 mm, and C: < 0.7 mm. Similarly, cancellous bone density was classified into levels 1, 2, and 3 based on the density from high to low. Furthermore, the GV value was converted into the absolute BMD value by the use of dental CBCT image calibration phantoms (Micro CT-HA phantom, QRM GmbH, Moehrendorg, Germany). The density classification intervals are 1: > 600 GV (= 420 g/cm^3^), 2: 300–600 GV (= 160 g/cm^3^–420 g/cm^3^), and 3: < 300 GV (= 160 g/cm^3^). According to categories of cortical bone thickness and cancellous bone density, the measurement result of the mean value and standard deviation for each bone type is shown in Table 1.

**Table 1:** Results of each bone type measurement in the new classification system

Mean value ± standard deviation		**Cortical bone thickness**		
		**A**	**B**	**C**
**Cancellous Bone Density**	**1**	1.39 ± 0.36	0.88 ± 0.11	0.57 ± 0.07
		781.85 ± 134.33	781.85 ± 134.33	781.85 ± 134.33
	**2**	1.39 ± 0.36	0.88 ± 0.11	0.57 ± 0.07
		452.71 ± 134.33	452.71 ± 134.33	452.71 ± 134.33
	**3**	1.39 ± 0.36	0.88 ± 0.11	0.57 ± 0.07
		198.79 ± 75.92	198.79 ± 75.92	198.79 ± 75.92

*Data presented as mean value* ± *standard deviation. Note: In each bone type, the upper row represents the cortical bone thickness value (unit: mm), and the lower row represents the cancellous bone density (unit: grayscale value, GV)*

### The percentage of nine bone types for the whole jawbone based on the new bone classification

According to the permutation and combination of different types of cortical bone thicknesses and cancellous bone densities, the analyzed jawbones were divided into nine classes: A1, A2, A3, B1, B2, B3, C1, C2, and C3 (Table 2), where the number of samples in each type and their proportions were detailed. As seen below, most of the jawbones at the potential implant sites were of intermediate quality. Among them, the most expected quality was B2, which was observed at up to 82 sites (26.03%), followed by A2 at 41 sites (13.02%), and C2 at 45 sites (14.29%). The least common was A3 at 13 sites (4.13%). In addition, it can be seen from the table that bone types C1 and A3 were barely mentioned in the previous literature, despite representing 4.44% and 4.13% of the samples collected in this study, respectively.

**Table 2:** The proportion of nine bone types for the whole jawbone is based on the new bone classification

Percent of total (Amount)		**Cortical bone thickness**		
		A	B	C
**Cancellous bone density**	1	8.57% (27)	17.78% (56)	4.44% (14)
	2	13.02% (41)	20.63% (65)	14.29% (45)
	3	4.13% (13)	8.57% (27)	8.57% (27)

**Fig. 5:**
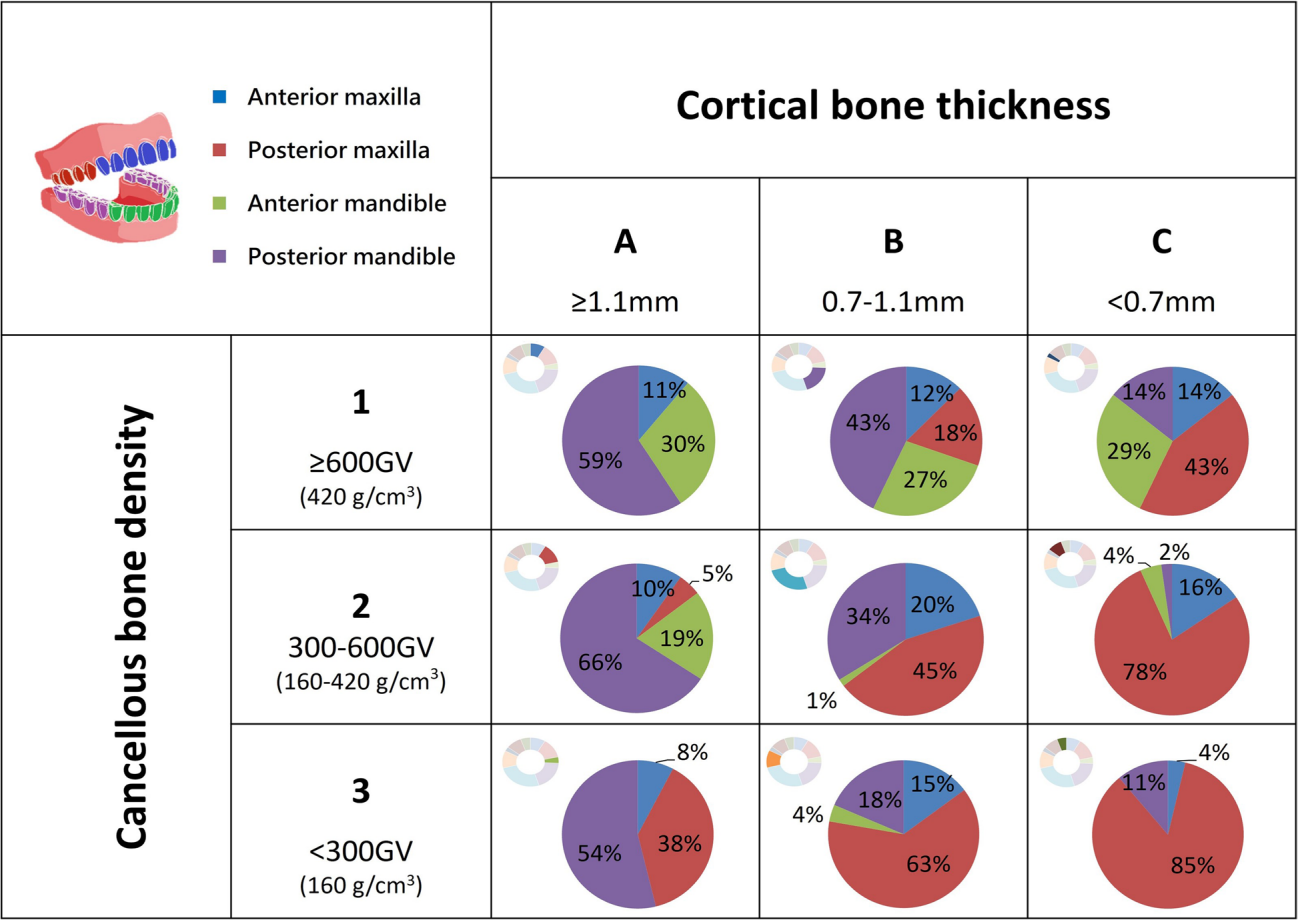
The constructions of the four jawbone regions for each bone type are based on the new bone classification

Furthermore, the proportions of all four regions (anterior maxilla, anterior mandible, posterior maxilla, posterior mandible) in each type were counted (Fig. 5). Type A1 was mainly found in the mandibular region of the jawbone (30% of the anterior mandible and 59% of the posterior mandible), which exhibits the greatest cortical bone thickness and the highest cancellous bone density. Type A3 has the greatest cortical bone thickness and the lowest cancellous bone density, 54% of which was found in the posterior mandible region. Type C1 has the lowest cortical bone thickness and the highest cancellous bone density, 43% of which was found in the posterior maxilla region. Type C3 represents the worst bone quality, which was mostly found in the maxilla region, where the posterior maxilla accounts for 85%.

### The percentage of bone types based on the four jawbone regions

**Fig. 6:**
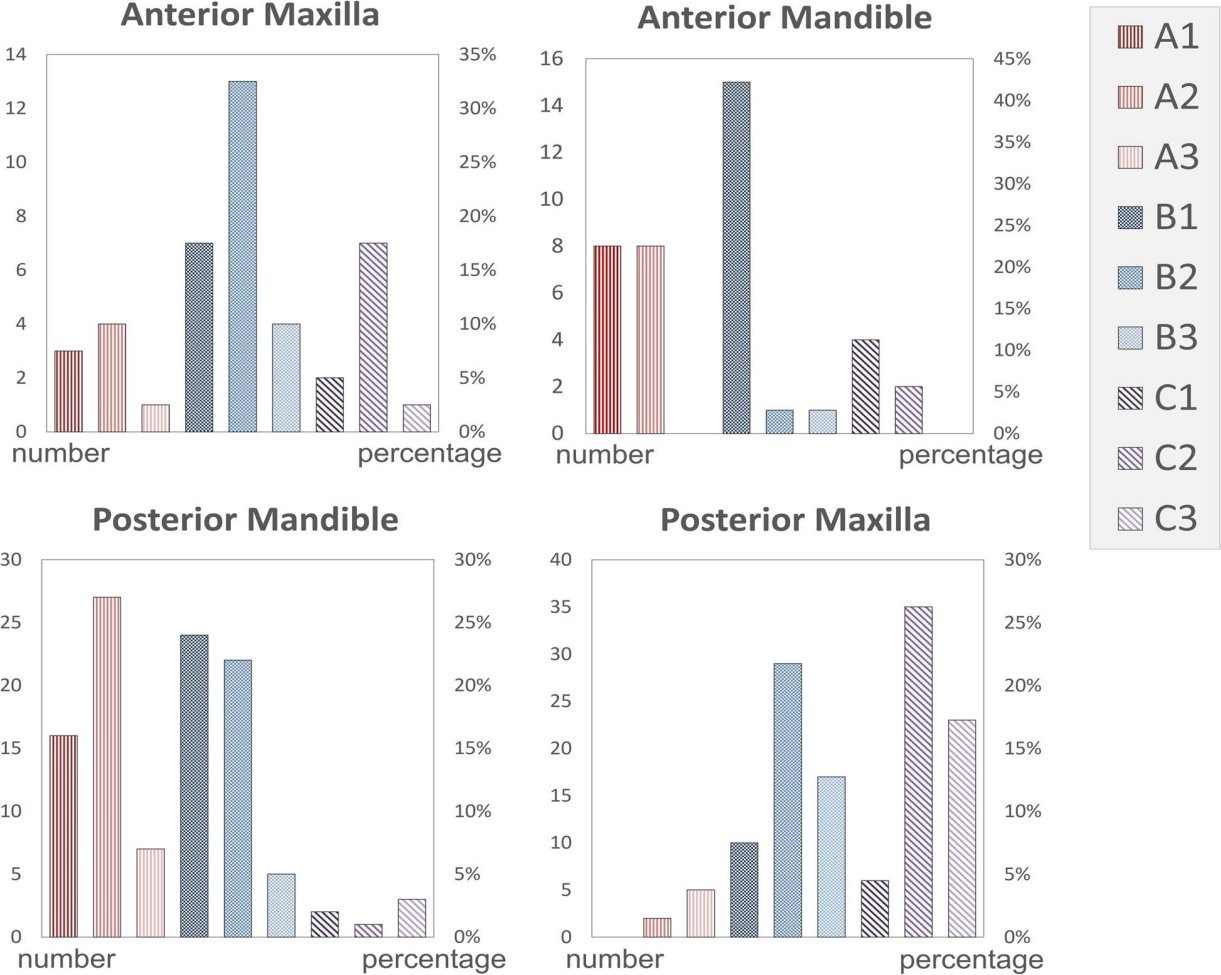
The case number of nine bone types in the four jawbone regions

Among the nine bone types obtained from the new bone classification in the four jawbone regions (Fig. 6), the overall bone types were mostly of intermediate quality in each region, with the exception of the posterior maxilla region. Grouping the results by region shows that the bone quality distribution in the anterior maxilla region follows the same pattern as the overall proportion. The relatively poor-quality bone types (B3, C2, and C3) in the posterior maxilla region accounted for 59.06% of the total, thus exhibiting a lower bone quality in comparison with the other regions. The bone qualities in the mandibular regions were generally better than those in the maxillary regions. Most of the bone types present in the anterior mandible region were A1, A2, and B1, and in this experiment, the C3 bone type was not found in this region. However, the posterior mandible region had more than 80% of A1, A2, B1, and B2 bone types.

## Discussion

The purpose of this study was to propose a new type of jawbone classification system. The previously held belief in the classification of jawbones was that the thicker the cortical bone, the denser the cancellous bone. In reality, this concept cannot accommodate all of the jawbone conditions [16] that may be present in clinical settings. According to previous research, cortical bone thickness and cancellous bone density play distinct roles in maintaining dental implant stability [17, 18]. Thus, when classifying jawbone quality, cortical and cancellous bones must be considered separately. Over the past few years, different combinations of jawbone thickness and density have been proposed [13]. However, cortical bone thickness and cancellous bone density are not quantitatively measured but only conceptually categorized by previously established classification systems. Given the aforementioned points, this study combined crestal cortical bone thickness and cancellous bone density measured by dental CBCT for quantitative analysis so as to provide a more comprehensive clinical and academic classification of jawbones. In addition, the various jawbone structures and the different ratios of cortical and cancellous bones, which may be present in clinical settings, were also simulated. Based on the measurement results, a new type of quantitative jawbone classification system has been established to meet the dental implant patient’s clinical needs and to provide reference information before and after dental implant surgery on bone quality classification.

The survival rate of dental implants depends on many factors [19, 20], one of which is bone quality and quantity. There have been many studies on the relationship between a patient’s jawbone quality and the success rate of dental implants [19, 21–23]. In the literature that explores the success rate of dental implants according to the different bone types, Jaffin et al. [22] followed the observation of a thousand dental implants and analyzing the results based on Lekholm and Zarb’s bone classification standard, they found that the failure rate for dental implants in Bone type IV sites was up to 35% but was only about 3% in total for Bone type I–III sites. Jemt et al. [24] also pointed out that the failure rate for implants after implantation in bone with better quality was only 7.9%, while that in bone with poor quality could reach 28.8%. Besides cancellous bone density, the cortical bone thickness at the edentulous site is another critical factor affecting the initial stability of a future implant. Miyamoto et al. [21] examined the initial implant stability in 225 implant sites using a resonance frequency analyzer. They found that the thicker the cortical bone is, the higher the initial implant stability would be. Song et al. [23] employed CBCT to assess the bone quality of the jawbone and measured the implant stability quotients (ISQ value) after the implantation of dental implants. They found that thicker cortical bone layers delivered higher dental implant stability.

Each previous jawbone classification method has its own pros and cons. As previously mentioned, the Type I method concerns Lekholm and Zarb’s classification system [8] and its extensions. At present, this is the most commonly used classification method in dental clinics. However, this method is subject to subjective judgment; thus, different observers may arrive at different conclusions and influence the experimental outcomes. Numerous scholars have therefore improved and extended the classification model of Lekholm and Zarb to classify the bone density of each bone type quantitatively. One of the representative studies was conducted in 2001 by Norton and Gamble [9], who employed CT to measure jawbone density. Despite the use of CT and CBCT images to quantify the bone density of each bone type in the aforementioned methods, the thickness of the cortical bone layer has not been considered in conjunction. Furthermore, in this type of classification concept, it is assumed that the cortical bone thickness and the cancellous bone density are positively correlated. However, the results of the present study show that the variation trends of cortical bone thickness and cancellous bone density are not consistent. In comparing previous literature on cortical bone thickness in the four jawbone regions [25–27] and on cancellous bone density [10, 14, 15, 28, 29], it can be seen that these two parameters exhibit different patterns. In addition, our team’s previous research further demonstrated that there is only a low correlation between the two [16] which indicates that in jawbones, “the thickest cortical bone may be paired with the most porous cancellous bone, and the thinnest cortical bone may also be paired with the densest cancellous bone.” In other words, Lekholm and Zarb’s classification system cannot accommodate all clinically possible conditions of bone qualities. The Type II bone classification method relies on the tactile sensation when drilling bones. The technique was originally developed by Misch et al., who used the tactile sensation from logging wood to simulate the tactile sensation of drilling bone during dental implantation [12]. However, clinicians and researchers might find this description relatively abstract. It was only recently that the Type III jawbone classification method was introduced. Tomaso Vercellotti suggested considering the cortical bone and the cancellous bone separately, where the cortical bone thickness is clearly defined, and the cancellous bone density is conceptually classified [13]. The growth thickness of cortical bone is measured at different time points after tooth extraction in this method of classification. Nevertheless, the amount of bone formation and the growth rate of bone differ in individuals in clinical settings. This method should be used with caution when describing the bone conditions of “different implant patients at the same time point” due to the slight error that may occur between this classification system and the actual bone thickness measurements.

In Lekholm and Zarb’s jawbone classification system and its extensions as aforementioned, cortical bone and cancellous bone are mostly considered as one unit to be classified, where it is assumed that the thicker the cortical bone, the denser the inner cancellous bone. However, in Tomaso Vercellotti’s classification method, the cortical bone thickness and the cancellous bone density of the jawbone are discussed separately, which is very different from the previous bone classification approach. In the system later proposed by Al Ekrish et al. [11], Types 2 and 3 are further subdivided according to the cancellous bone density from high to low, implying that cortical bone and cancellous bone may need to be treated separately when classifying bone quality. In the previous studies focused on the cancellous bone density at dental implant sites [14, 15], generally, it has been noted that cancellous bone density is the highest in the anterior mandible, followed by the anterior maxilla, posterior mandible, and posterior maxilla. However, in the previous studies focused on the cortical bone thickness at dental implant sites [25, 27], the posterior mandible had the thickest cortical bone, followed by the anterior mandible, anterior maxilla, and posterior maxilla. This indicates that the cancellous bone density and cortical bone thickness in the four jawbone regions. Moreover, the previous research performed at our laboratory also demonstrated a low correlation, or even no correlation in some jawbone regions, between the cortical bone thickness and the cancellous bone density [16], which suggests that the two bone structures play different roles in maintaining the stability of dental implants [17, 18]. Therefore, it is believed to be necessary to consider the cortical and cancellous bones separately in the classification of jawbones.

Cortical bone thickness in the potential dental implant sites was measured in the central cross-section of the radiographic guides, following the measurement proposed by Ko et al. [27] and Gupta et al. [25]. In addition, Wang et al. used the same measurement method [16]. From the research above, the thickness of the crestal cortical bone is within 2 mm (mostly between 0.7 and 1.2 mm) [16, 25, 27]. This value is significantly lower than the buccal and lingual cortical bone thicknesses (approximately 1–3 mm) [30, 31] due to the crestal cortical bone growth after tooth extraction. Therefore, the interval of thickness classification is bound to differ from that of other areas (buccal and lingual) to better represent the actual situation in this area. Previous studies have proposed using 1 mm as the boundary, and the cortical bone will be considered “thick” when the thickness is greater than 1 mm [32]. In addition, Salimov et al. scored bone quality by the tactile sensation of cortical and cancellous bone by surgeons during dental implants. This study identified a thick cortical bone ≥1 mm [33]. Previous research has pointed out that resistance in the early drilling stage is closely related to the cortical bone [34, 35]. Linck et al. [36] classified bones according to tactile sensation during bone drilling in the literature on surgeons' tactile sensation and cortical bone. The results showed that the worst bones (easy to insert implants) were approximately 23%. In addition, Alsaadi et al. studied the correlation between tactile and stability parameters during surgery [37]. The results showed a high correlation between PTV and the surgeon's tactile sensation with the worse cortical bone in the group accounting for about 23% (10/44). To prevent inconvenience in clinical applications, this study did not make detailed divisions of the jawbone density and thickness. Therefore, the cancellous bone density and cortical bone thickness in the new bone classification system are only divided into three categories (Density: 1, 2, 3; Thickness: A, B, C; values from high to low). In the present study, the Class C cortical bone was about 25%, a result that is similar to the previous research. According to the research mentioned above, the three categories of cortical bone thickness in this research are as follows: A, >1.1 mm; B, 0.7–1.1 mm; and C, <0.7 mm.

**Fig. 7:**
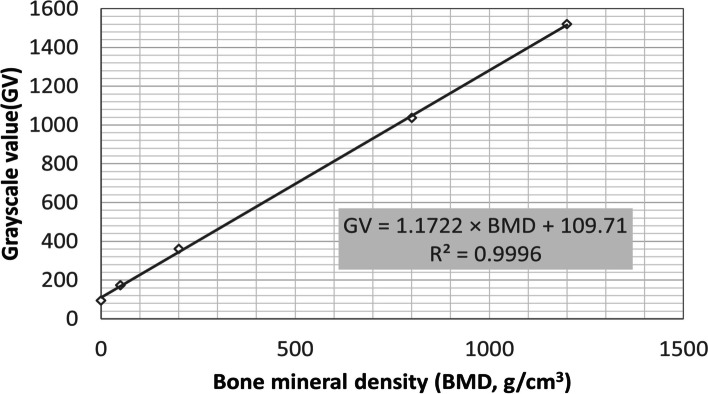
The relationship between bone mineral density (BMD) and grayscale value (GV). The transfer formula between BMD and GV is also listed in this figure

While setting the boundaries for high, intermediate, and low cancellous bone densities, this study referred to many previous publications on the jawbone densities of dental implant sites. Alkhader et al. [38] studied the CBCT images of the molar and premolar regions before dental implantation. The jawbones were classified as low-, intermediate-, and high-density by two experienced observers. They found that low-density bones made up 15.6% of the total, intermediate-density 47.9%, and high-density 36.5%. Their results revealed that bones of intermediate density are the most common type, followed by those of high density, and the low-density ones are the least common. Dahiya et al. [39] also obtained similar results. They found that in the same molar and premolar regions, low-density bones made up 21% of the total, intermediate-density 39.5%, and high-density 39.4%; the average jawbone density for women was 580.2 ± 120.22 GV, which was below the average of 690.5 ± 104.12 GV for men. According to the previous literature, when setting the CBCT measurement value of > 600 GV as high density, 300–600 GV as intermediate density, and < 300 GV as low density in this study, the respective proportions in the posterior mandible are: low-density 14.01%, intermediate-density 46.73%, and high-density 39.25%. These results are similar to those reported in the previous literature (Fig. 7), such that in this region, the proportion of intermediate-density bones is the greatest, and that of low-density bones is the least. According to this definition, in the whole jawbone, high density is about 30%, intermediate density is 45%, and low density is 25%. Also, the proportion of each density level is highly consistent with the research results of Alkhader et al. [38]. In addition, it can also be found that the average jawbone density in the posterior mandible is 535 ± 206 GV, which is slightly lower than that reported by Dahiya et al. [39] This is probably due to the fact that dental CBCT’s value is likely to vary depending on the brand, the equipment, etc. Furthermore, the average age of subjects in this study is 52.1 years, whereas the average age of subjects in the study by Dahiya et al.is 42.5 years. Many previous studies have indicated that there is a positive correlation between age and the loss of jawbones [40, 41].

In this quantitative jawbone classification system, the B2 type is the most common bone type, accounting for 20.63% of the total (65 sites). A3 type (i.e., the thickest cortical bone with the loosest cancellous bone) is the least observed bone type, only accounting for 4.13% of the total (13 sites). In the past, classification systems generally believed that the cortical bone thickness and cancellous bone density should be positively correlated; that is, the thicker the cortical bone is, the denser the cancellous bone density will be, and vice versa. This concept, however, is only partially accurate. There may be different combinations of cancellous bone density and cortical bone thickness. In a paper previously published by our laboratory, only a low correlation was observed between jawbone thickness and density [16]. Moreover, this finding was further confirmed by the results of the current study. From the actual measurements, C1 (the thinnest cortical bone with the densest cancellous bone) and A3 (the thickest cortical bone with the most porous cancellous bone) can also be observed at the potential dental implant sites in patients. Although they are not the most prevalent jawbone qualities in clinical settings, they still represent 4.44% (C1) and 4.13% (A3) of the total, respectively. In the commonly used jawbone classification systems such as Lekhlom and Zarb, the A3 and C1 bone types have yet to be described, but the results of this experiment suggest they account for approximately 10% of all imaging samples of potential dental implant sites. Therefore, the purpose of this paper is to develop a new type of jawbone classification system to better meet the clinical needs and complement the parts overlooked in previous bone classification methods (bone quality such as A3 and C1). Additionally, it can be used in conjunction with postoperative tracking to analyze the initial stability of dental implants in bones of different qualities and their long-term success rate.

**Fig. 8:**
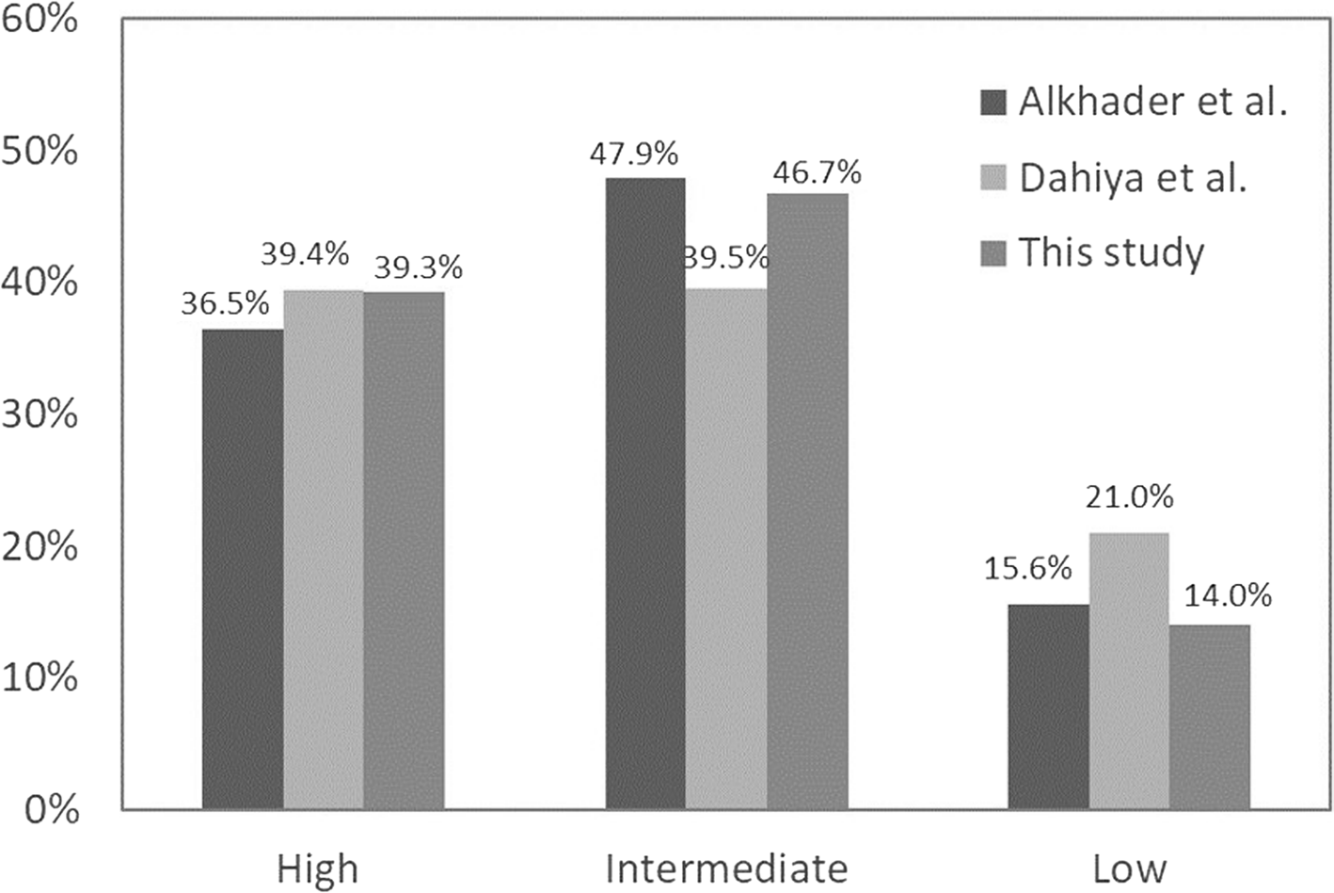
Percentage of low-, intermediate- and high-density in the posterior mandible region compared with previous studies [42, 43]

In the literature related to dental implants, Liu et al. [42] employed dental CBCT to assess jawbone density to determine whether this technique would be appropriate for evaluating treatment plans for dental implants. Their research results demonstrated that CBCT images could provide valid information on jawbone density and other bone quality characteristics, making it a very appropriate assessment tool before dental implant surgery. The GV obtained from dental CBCT is not a real HU; however, its working principle is also based on the linear relationship between the radiation absorption and the object density, where objects of different densities are presented with different GVs. In this sense, it is also suitable for evaluating jawbone density. Nomura et al. [43] once pointed out that although the GV of dental CBCT imaging would be higher than the HU of traditional CT imaging, these two values are still positively correlated with bone density. Using dental CBCT GVs to indicate the density of test objects was deemed inaccurate in some previous literature [44, 45]. According to them, even though the image GVs of CBCT are linear, the absolute values can be easily affected by factors such as voltage (kVp), current (mAs), and the different instrument manufacturers. In this study, the results of cancellous bone density were standardized from GV to BMD(g/cm^3^). In this manner, the image GVs were converted into actual BMD values (g/cm^3^) (Fig. 8), which are also listed in the research results, thereby providing a reference basis for future scholars to cite our method. With this conversion, even with different brands of CBCT, users of this new jawbone classification system will be able to convert between the two values by scanning the BMD phantom to obtain the image GV. This way, future clinical use will not encounter errors arising from the use of different CBCT machines, thereby facilitating future clinical applications.

For the clinical applicability of the new method proposed in this study, dentists should pay more attention to choosing appropriate dental implants or preparing the host bone for the A3 (thick cortical bone and low-density cancellous bone) and C1 (thin cortical bone and high-density cancellous bone) implant positions before dental implant surgery. Postoperative follow-up of the 315 implants in this study will be studied in the future. We hope to provide clearer recommendations to dentists in selecting dental implants or surgical techniques corresponding to the nine different bone states for the new classification method proposed in this study.

As far as the limitations of this study are concerned. Even though the sample size of this study is more significant than that of similar retrospective studies [9, 10, 14, 15, 28], in the future, if it is necessary to conduct subgrouping studies based on different sexes, ages, and implant sites, more dental implant samples should be collected to represent the entire population, in addition, this study only discussed the dental CBCT images of patients with dental implants before the surgery and did not follow up on these patients after the dental implantation. In the future, the survival rate of these dental implants in these patients should be tracked, and its correlation with the bone type should be explored.

## Conclusions

In conclusion, the proposed bone classification is a new quantitative classification system of jawbone quality and quantity at the dental implant site developed based on dental CBCT. In this system, the bone quality and bone quantity of the jawbone are classified into nine bone types. That is, the crestal cortical bone thickness is classified into A: > 1.1 mm, B: 0.7–1.1 mm, and C: < 0.7 mm, and the cancellous bone density is classified into 1: > 600 GV (= 420 g/cm^3^), 2: 300–600 GV (= 160–420 g/cm^3^), and 3: < 300 GV (= 160 g/cm^3^). The proposed classification system verified that nine possible types of bone were found in all maxilla and mandible regions.

## Data Availability

The datasets used and/or analysed during the current study are available from the corresponding author on reasonable request.
